# The *F1000Research:* *Ebola* article collection

**DOI:** 10.12688/f1000research.5685.1

**Published:** 2014-11-07

**Authors:** Peter Piot

**Affiliations:** 1 London School of Hygiene & Tropical Medicine, Keppel Street, London, WC1E 7HT, UK

## Abstract

The explosion of information about Ebola requires rapid publication, transparent verification and unrestricted access. I urge everyone involved in all aspects of the Ebola epidemic to openly and rapidly report their experiences and findings.

**Editorial**

In addition to all the formidable challenges we are confronting with the Ebola epidemic, the information challenge is of no less significance than all the others. Within the exploding growth of the epidemic itself there is an explosion of information that requires rapid publication, transparent verification and unrestricted access. This information includes organisational and logistical experiences, transmission narratives, presentation, methods of recognition, case histories, a range of orthodox and unorthodox interventions, clinical trials, biological and virological findings, and many more.

*F1000Research* is a publishing platform that combines immediate publication, transparent refereeing, and unrestricted open access, making it extremely suited to respond to these information challenges. *F1000Research*, has offered to publish free of charge a collection of reports and publications on all issues relating to the Ebola epidemic. All papers published by *F1000Research* are open access. I urge everyone involved in all aspects of this epidemic to openly and rapidly report their experiences and findings. Information will be one of our key weapons in defeating the Ebola epidemic.

